# Dietary inclusion of *Pleurotus ostreatus* spent mushroom substrate in corn silage-based diets: effects on rumen fermentation, nutrient digestibility, and greenhouse gas emissions using a RUSITEC system

**DOI:** 10.1007/s11356-026-37792-y

**Published:** 2026-05-05

**Authors:** Chika C. Anotaenwere, Omoanghe S. Isikhuemhen, Peter A. Dele, Ahmed E. Kholif, Kiran Subedi, Michael Wuaku, Joel O. Alabi, Oludotun O. Adelusi, Kelechi A. Ike, Deborah O. Okedoyin, DeAndrea Gray, Uchenna Y. Anele

**Affiliations:** 1 Department of Animal Sciences, North Carolina Agricultural and Technical State University, Greensboro, NC 27411 USA; 2 Department of Natural Resources and Environmental Design, North Carolina Agricultural and Technical State University, Greensboro, NC 27411 USA; 3 Department of Pasture and Range Management, Federal University of Agriculture, Abeokuta, Ogun State Nigeria; 4 Dairy Science Department, National Research Centre, 33 Bohouth St., Dokki, Giza, Egypt; 5 Analytical Services Laboratory, North Carolina Agricultural and Technical State University, Greensboro, NC 27411 USA

**Keywords:** Corn silage, Methane emissions, RUSITEC, Spent mushroom substrate, Rumen fermentation

## Abstract

This study evaluated the impact of incorporating *Pleurotus ostreatus* spent mushroom substrate (SMS) into corn silage-based diets on rumen fermentation, fiber digestibility, and biogas emissions using the Rumen Simulation Technique (RUSITEC). Given the need to reduce feed costs and mitigate environmentally harmful emissions from rumen fermentation, SMS was evaluated as a partial replacement for corn silage at inclusion levels of 10% (T1), 20% (T2), and 40% (T3). These three treatments were compared to a control diet consisting of 100% corn silage, to assess rumen fermentation characteristics, nutrient digestibility and biogas emissions, thereby determining the feasibility of SMS as a functional feed component in sustainable ruminant production systems. Significant improvements (*P* < 0.001) were observed in dry matter digestibility, which increased from 41.1% in the control to 46.8% and 48.1% in the T1 and T2 treatments, respectively. Likewise, neutral detergent fiber digestibility rose from 56.7% (control) to 62.3% (T1) and 65.1% (T2). SMS inclusion significantly decreased methane (CH_4_) emissions (*P* < 0.001), with the 10% SMS treatment reducing CH_4_ from 65.6 to 14.4 mg/g DM, a reduction of about 78%. Ammonia levels also declined significantly (*P* < 0.001) from 1025 mmol/g DM in the control to 420 mmol/g DM in the T1 group. Hydrogen sulfide emissions showed a similar pattern (*P* < 0.001), dropping from 7828 mmol/g DM (control) to 1817 mmol/g DM (20% SMS). Although total volatile fatty acids were not significantly affected, acetate levels increased (*P* = 0.046) to 74.9% (T2), and valerate was significantly higher (*P* < 0.001) in the T1 group (2.58%). These results indicate that replacing 10–20% of corn silage with *P. ostreatus* SMS can significantly enhance nutrient digestibility and reduce environmental emissions, without affecting fermentation characteristics. SMS is an economical, eco-friendly, and promising feed additive for sustainable ruminant farming.

## Introduction

The rising costs of livestock feed ingredients, along with the growing environmental impacts associated with livestock farming, have escalated the demand for more sustainable and cost-effective feeding strategies. However, increasing concerns related to feed costs and environmentally harmful emissions arising from rumen fermentation have intensified interest in alternative feed resources that can maintain productivity while improving environmental sustainability. Corn silage has inherent limitations, such as low crude protein levels and suboptimal fiber digestibility, that restrict its use as the primary forage for high-producing ruminants (Krause and Combs 2003). These limitations increase reliance on costly protein supplements and reduced feed efficiency. In addition, rumen fermentation of conventional forage-based diets contributes substantially to greenhouse gas emissions, particularly CH4, underscoring the importance of identifying functional feed components that can mitigate emissions without compromising rumen function. These challenges necessitate accessible, nutritious, and environmentally sustainable alternative feed resources.

Recent studies have found agro-industrial byproducts rich in nutrients that can be fermented at a low cost and are easily accessible, offering potential solutions. One promising alternative feed source is spent mushroom substrate (SMS), the leftover material from growing *Pleurotus ostreatus*. The utilization of SMS in ruminant nutrition has been extensively investigated, particularly in South Asian countries, where mushroom production and SMS availability are substantial (Mallick and Mukherjee Sanyal 2023). This substrate is primarily composed of lignocellulosic residues, often characterized by relatively high lignin and polyphenolic compounds, including condensed tannins, in addition to residual fungal enzymes, partially degraded fibers, and nitrogen compounds. While lignin and polyphenols may reduce fiber digestibility and protein availability, they are also associated with reduced rumen protozoal populations and methanogenic activity, leading to lower CH_4_ production. Incorporating SMS into livestock diets can reduce feed costs because of its low economic value while simultaneously supporting sustainable waste management in the mushroom industry, thereby contributing to a circular bioeconomy (Anotaenwere et al. 2024). Additionally, diverting SMS from landfills and incineration reduces the environmental footprint of mushroom production.

Methane emissions have been shown to decrease through the supplementation of *P. ostreatus* SMS in ruminant diets (Anotaenwere et al. 2024). These changes result from shifts in fermentation patterns and improved microbial efficiency in the rumen. SMS could alter the balance of volatile fatty acids (VFAs) in the rumen, increasing propionate production and reducing methanogenesis. Thus, dietary manipulation using SMS represents a promising strategy to enhance fermentation efficiency and support climate-resilient livestock production (Anotaenwere et al. 2024).

Previous studies have shown that *Pleurotus* species can effectively break down lignin, which enhances the digestibility and nutritional value of crop residues like corn stover (Song et al. 2020). This effect is primarily mediated through fungal ligninolytic enzymes, including laccases and manganese peroxidases, which disrupt lignin-carbohydrate complexes and increase the accessibility of cellulose and hemicellulose to rumen microorganisms (Wuaku et al. 2025). In addition, partial delignification during mushroom cultivation reduces physical barriers within the plant cell wall, facilitating microbial attachment and enzymatic hydrolysis in the rumen (Kholif et al. 2022). Recently, Anotaenwere et al. (2024) studied the effect of *P. ostreatus* SMS inclusion on fiber digestibility using in vitro batch culture system. These improvements are likely linked to enhanced fibrolytic activity and shifts in microbial fermentation pathways, although confirmation under semi-continuous rumen conditions remains limited. However, most studies have relied on short-term in vitro batch fermentation systems (Olagunju et al. 2023); however, in vitro systems do not fully reveal the continuous dynamics of fermentation and microbial adaptation observed under in vivo conditions.

The Rumen Simulation Technique (RUSITEC) is a semi-continuous in vitro model that mimics rumen dynamics in a controlled environment, enabling repeated sampling and long-term monitoring of fermentation parameters, digestibility, and activity of microbes. Unlike batch culture, RUSITEC maintains steady fermentation conditions over extended periods, better reflecting the in vivo ruminal environment (Wetzels et al. 2018). The RUSITEC system provides a dynamic fermentation environment with continuous buffer infusion and removal of fermentation end-products, minimizing accumulation effects and maintaining stable pH and microbial activity (Shaw et al. 2023).

This study hypothesizes that the addition of different levels of *P. ostreatus* SMS to corn silage would enhance digestibility and economic performance, reduce GHG emissions, and maintain the feed’s nutritional value. Accordingly, this study used the RUSITEC system to evaluate the effects of SMS inclusion at 10%, 20%, and 40% to corn silage on rumen fermentation, fiber digestion, and GHG production.

## Materials and methods

### Study ethical approval

North Carolina Agricultural and Technical State University’s Institutional Animal Care and Use Committee (IACUC) approved all animal procedures for this study. The procedures were carried out at the university’s Beef Research and Training Facility (BRTF) in Greensboro, NC, USA. According to protocol LA21-009, the BRTF performed daily health checks on the cannulated cows and provided treatment following standard management practices.

### Silage preparation

The preparation of silage followed the method described by Anotaenwere et al. (2024). Corn was hand-harvested from the university farm, leaving a 15-cm stubble, once the kernels reached about one-third milk line maturity. The harvested corn was then chopped into pieces about 2 cm long using a forage chopper. Spent mushroom substrate, made entirely from corn stover (100% of the growth medium), was produced in the mycology laboratory. For the ensiling, a 5-L cylindrical plastic bucket of 35 cm high and 16 cm wide was used to ensile blends of corn and SMS, and each storage duration had four replications. Ensiling was carried out within 6 h of harvesting, and the buckets were stored at room temperature (20 ± 2 °C) for 42 days. Both after filling and when opened, silos were weighed to determine dry matter (DM) recovery. After opening, samples were collected for chemical composition analysis.

### Study design and chemical analysis

The study used a completely randomized design with four replicates per treatment, including sole whole-crop corn silage (whole corn plant including grain) as the control, 90% whole-corn (including grain) + 10% SMS ensiled for 42 d (T1), 80% whole-crop corn (including grain) + 20% SMS ensiled for 42 d (T2), and 60% whole-crop corn (including grain) + 40% SMS ensiled for 42 d (T3). These dietary treatments were formulated based on results from a previous study (Anotaenwere et al. 2024). Therefore, in this study, treatments with low (10%), moderate (20%), and high (40%) inclusion levels were chosen for further testing using the Rumen Simulation Technique (RUSITEC). These inclusion levels were selected to represent increasing replacement of whole-crop corn by SMS while maintaining ensiling feasibility and nutritional balance, and were considered optimal based on a balance of improved fermentability, fiber utilization, and environmental benefits recorded in the previous batch culture experiment.

For this study, all data were analyzed using AOAC (2019). Standard procedures were followed for ether extract, DM, ash, and crude protein (CP; nitrogen × 6.25). Calculations were performed for organic matter (OM) by the subtraction of ash content from the DM, which is expressed on percentage basis. ANKOM 200 Fiber Analyzer (ANKOM Technology Corporation, Fairport, NY, USA) was used to analyze acid detergent fiber (ADF; Method #973.18). The determination of Neutral Detergent Fiber (NDF) was implemented according to the procedure outlined by Van Soest et al. (1991). Acid detergent lignin (ADL) was measured by solubilizing cellulose with concentrated sulfuric acid, following the analytical protocols provided by ANKOM Technologies. Hemicellulose content was determined by deducting ADF from NDF, while cellulose content was estimated by subtracting ADL from ADF. Nonfibrous carbohydrates (NFC) were calculated as 100 − NDF − CP − EE – ash.

### RUSITEC fermentation

The RUSITEC system included two identical fermenters, each with eight vessels (total of 16), each maintained at 39 °C and continuously infused with artificial saliva buffer under anaerobic conditions. These chambers had 1000-mL fermentation vessels, which were randomly assigned to four groups, each with four replicates. Each vessel is comprised of an inlet for adding artificial and an outlet for the collection of effluent. At the start of the experiment, 700 mL of rumen fluid and 200 mL of artificial saliva were poured into each of the vessels, adhering to the buffer recipe specified by McDougall’s (McDougall 1948): (0.60 g/L KCl, 9.83 g/L NaHCO_3_, 3.69 g/L Na_2_HPO_4_, 0.47 g/L NaCl, 0.30 g/L (NH4)2SO4, 0.061 g/L MgCl_2_.6H_2_O, and 0.0293 g/L CaCl_2_.2H_2_O). Following an initial adaptation phase, nylon bags containing the experimental diets were incubated in each vessel and replaced every 24 h to simulate rumen retention time. The rumen fluid had an initial pH level of 6.61, while the pH of artificial saliva measured 8.54.

Three black Angus beef cows, each fitted with a rumen cannula and weighing 550 ± 10 kg, served as donors for the rumen inoculum in the RUSITEC fermenters. The cows had unlimited access to water and pasture, which mainly consisted of hay and grass (over 99% forage) and a mineral supplement of less than 1%. Rumen contents were gathered from the ventral, dorsal, and cranial regions of the cannulated animals. Rumen fluid from three cows used for this study was mixed, sieved through four layers of cheesecloth, and delivered to the lab in a Thermoflask. Fifty grams (50 g) of rumen solids were added to each vessel on the first day of incubation and allowed to sit for 24 h before being removed. The substrates were weighed, stored in sample bags and kept in the vessels for 48 h of fermentation. A water bath of 39 °C was used to submerge the fermenters with an infusion of continuous artificial saliva which was maintained at 21 rpm using a Watson-Marlow Pump 205U. The whole experiment was carried out in 9 days, which had two phases of 4 days for adaptation and 5 days for the collection of data.

### Fermentation measurements

During the 5-day sampling period, daily gas produced was measured in milliliters per day (mL/d) and collected in a Tedlar gas sampling bag (Supelco, Bellefonte, PA, USA) connected to the effluent flasks. Gas pressure readings were carried out daily using a gas flowmeter (DM3, Alexander Wright Ltd., London, UK). A portable gas analyzer (Biogas 5000, Landtec, Dexter, MI, USA) measured GHG, including CH_4_, ammonia (NH_3_), CO_2_, and hydrogen sulfide (H_2_S). This analyzer has an internal electrochemical and dual-wavelength infrared sensors with a reference channel. The analyzer was calibrated, and gas readings were taken by connecting the device to the gas collection opening on each effluent flask. This was performed in accordance with the manufacturer’s instructions. For accuracy, the unit was purged after each sampling to remove any residual gas from the previous measurement.

The volume of effluent was measured daily using a graduated cylinder when the feed bag was changed. Measurement of pH were immediately determined with a Fisher brand pH benchtop meter (Fisher Scientific, Waltham, MA, USA). For determination of VFA, preservative of 3 mL of 25% metaphosphoric solution was prepared and 15 mL of liquid effluent was collected, and immediately kept in − 20 °C freezer until needed for VFA determination. Gas chromatography (Agilent 7890B GC system with a Flame Ionization Detector and 7693 autosampler, Agilent Technologies, Santa Clara, CA, USA) and a capillary column (Zebron ZB-FFP, Phenomenex Inc., Torrance, CA, USA) were used to measure the Concentrations of VFA. A total of 20 replicate samples were collected for each treatment. Samples collected over the 5-day period were pooled by treatment prior to chemical analysis to obtain a representative composite sample and to reduce day-to-day analytical variability.

### Nutrient digestibility

Using the same sampling period mentioned earlier, sample bags were taken out of each fermenter after 48 h of fermentation. They were then washed with cold water until the water ran clear and oven-dried at 55 °C for 72 h. The DM digestibility (DMD) was estimated using the weight of the remaining residue. A known quantity of residue from each sample was burned using a muffle furnace at 550 °C for 3 h in crucibles and weighed to determine OM digestibility (OMD). Neutral detergent fiber (NDF), acid detergent fiber (ADF), and acid detergent lignin (ADL) were analyzed using residues from each bag with the Ankom Fiber Analyzer (ANKOM Technology, Macedon, NY, USA). These values enabled the calculation of their degradability (NDFD, ADFD, and ADLD, respectively).

### Statistical analysis

The Shapiro–Wilk test was employed to evaluate the normality of variables. Individual RUSITEC chambers (vessels) were considered the experimental units and randomly assigned to dietary treatments across two identical fermenters operating concurrently under identical controlled conditions. All variables exhibited a normal distribution. Analyses were performed using a completely randomized design in SAS (version 9.4; SAS Institute Inc., Cary, NC, USA) with the GLM procedure. Tukey range test was used to separate means of significant variables at *P* ≤ 0.05. The statistical model used in this study was *Y*_ij_ = *µ* + *T*_i_ + *e*_ij_, where *Y*_ij_ is the dependent variable, *µ* represents the overall mean, *T*_i_ stands for the treatment effect, and *e*_ij_ represents the residual error. Because treatments were evenly distributed among fermenters, all chambers received the same inoculum source, buffer infusion rate, temperature and management, and no systematic fermenter- or chamber-specific effects were observed, chamber-to-chamber variation was considered random and incorporated within the residual error term. Under these conditions, a one-way ANOVA model is appropriate and has been widely used in RUSITEC-based fermentation studies.

## Results

### Nutrient concentration

Table 1 shows the chemical composition (% DM basis) of diets with increasing proportion of *P. ostreatus* SMS replacing CS at 10, 20, and 40% in T1, T2, and T3, respectively, compared to a 100% corn silage control. The values represent analyzed composition after 42 d of ensiling and therefore reflect fermentation-induced changes rather than calculated mixture proportions. Dry matter content was not significantly affected by SMS inclusion (*P* = 0.301), with T1 showing a lower OM concentration compared to the control and T2. Crude protein content reached its peak in T3 (16.0%) compared to T1 (5.9%) (*P* = 0.023). Although EE content varied among diets, the differences were not statistically significant (*P* = 0.200). Non-structural carbohydrates declined significantly in T1 and T3 treatments (*P* = 0.001). Notably, the inclusion of SMS resulted in significant increases in fiber fractions, with NDF (in T1 only) and ADF rising substantially across treatments (*P* < 0.001). Acid detergent lignin also increased significantly (*P* < 0.001), particularly in T1, which showed the highest ADL content (7.37%). Hemicellulose content was significantly higher (*P* = 0.003) in T1, while cellulose showed a numerical increase but did not reach statistical significance (*P* = 0.072).

**Table 1:** Chemical composition (%, DM basis, except DM which is expressed on an air-dry basis) of diets with different spent mushroom substrate (SMS) and corn silage (CS) proportions

Treatments^1,2,3^	DM	OM	CP	EE	NFC	NDF	ADF	ADL	HC	CL
SMS	94.9	91.3^b^	4.4^b^	5.2	18.5^c^	63.1^a^	42.7^a^	11.5^a^	20.4^a^	31.2
Control	97.8	94.6^a^	9.8^ab^	2.60	42.9^a^	42.0^c^	26.3^d^	2.66^d^	15.7^b^	23.6
T1	98.1	91.8^b^	5.9^b^	1.59	33.9^b^	52.0^b^	34.7^b^	7.37^ab^	17.3^a^	27.3
T2	98.3	93.1^ab^	9.6^ab^	2.00	37.6^ab^	45.9^c^	30.0^c^	5.68^b^	15.9^b^	24.4
T3	97.5	93.3^ab^	16.0^a^	2.17	31.6^b^	45.6^c^	29.1^c^	4.02^c^	16.5^ab^	25.1
SEM	0.49	0.47	1.54	0.21	1.63	0.45	0.43	0.21	0.19	0.48
*P*-value	0.301	0.031	0.023	0.200	0.001	< 0.001	< 0.001	< 0.001	0.003	0.072

*ADF* acid detergent fiber, *ADL* acid detergent lignin, *CL* cellulose, *CP* crude protein, *DM* dry matter (air-dry basis), *EE* ether extract,* HC* hemicellulose, *NDF* neutral detergent fiber, *NFC* non-fibrous carbohydrates, *OM* organic matter, *SMS* spent mushroom substrate

^1^Treatments: Sole corn silage as the control, 90% corn silage + 10% SMS ensiled for 42 d (T1), 80% corn silage + 20% SMS ensiled for 42 d (T2), and 60% corn silage + 40% SMS ensiled for 42 d (T3)

^2^Values represent analyzed composition of the ensiled diets after 42 d and may differ from calculated pre-ensiling proportions due to fermentation-induced changes in nutrient fractions

^3^Previously reported byAnotaenwere et al. (2024)

Means with different superscripts within the same column differ, *p* < 0.05

### pH, effluent volume, and digestibility

The pH, and OMD were not significantly affected by the inclusion of SMS (Table 2). However, the T1 and T2 diets exhibited the highest (*P* < 0.001) DMD. With no differences compared to the control, T2 showed the highest (*P* = 0.042) volume of effluent compared to T1.

**Table 2:** Effects of partial replacement of corn silage with varying proportions of spent mushroom substrate on fermentation pH, volume of effluent, and in vitro nutrient digestibility

Treatments^1^	pH	Volume of effluent (mL/g substrate)	OMD (%)	DMD (%)	NDFD (%)	ADFD (%)	ADLD (%)
Control	7.44	555^ab^	88.2	41.1^b^	56.7^b^	42.7^b^	25.0^a^
T1	7.32	525^b^	90.8	46.8^a^	62.3^a^	41.8^b^	20.2^b^
T2	7.45	558^a^	86.9	48.1^a^	65.1^a^	49.3^a^	24.9^a^
T3	7.44	537^ab^	87.2	42.1^b^	63.8^a^	45.0^b^	19.4^b^
SEM	0.043	9.0	1.46	1.09	1.18	1.21	1.21
*P*-value	0.110	0.042	0.243	< 0.001	< 0.001	0.003	0.001

*OMD* organic matter digestibility, *DMD* dry matter digestibility, *NDFD* neutral detergent fiber digestibility, *ADFD* acid detergent fiber digestibility, *ADLD* acid detergent lignin digestibility

^1^Treatments included: Control (100% corn silage), T1 (90% corn silage + 10% SMS), T2 (80% corn silage + 20% SMS), and T3 (60% corn silage + 40% SMS)

Means with different superscripts within the same column differ, *p* < 0.05

Diets containing SMS significantly increased (*P* < 0.001) NDFD compared to the control. Furthermore, ADFD was highest (*P* = 0.003) in T2 (49.3%), while T1 and T3 showed (*P* = 0.001) lower values for ADLD compared to the control diet (Table 2).

### Volatile fatty acid profiles

Total VFA production did not significantly differ among treatments (Table 3). Acetate proportion was significantly affected (*P* = 0.046), with T2 yielding the highest value (74.86%) compared to T1 (69.6%), with no differences observed relative to the control. Valerate proportions increased significantly (*P* = 0.008) in T1 compared to T2 and T3, with no differences compared to the control. The acetate-to-propionate (A:P) ratio was not significantly affected.

**Table 3:** Effects of partial replacement of corn silage with varying proportions of spent mushroom substrate on total concentration and molar proportions of volatile fatty acids (VFA)

Treatments^1^	Total VFA (mmol/L)	Acetate (%)	Propionate (%)	Acetate:propionate	Butyrate (%)	Isobutyrate (%)	Valerate (%)	Isovalerate (%)
Control	56.7	71.9^ab^	14.4	5.26	11.3	0.39	1.78^ab^	0.21
T1	53.8	69.6^b^	15.6	4.67	11.6	0.39	2.58^a^	0.21
T2	52.5	74.9^a^	13.1	5.81	10.1	0.37	1.42^b^	0.19
T3	51.1	71.3^ab^	15.7	4.69	10.8	0.34	1.61^b^	0.18
SEM	1.96	1.36	0.86	0.362	0.57	0.041	0.235	0.024
*P*-value	0.155	0.046	0.115	0.101	0.282	0.789	0.008	0.685

^1^Treatments included: Control (100% corn silage), T1 (90% corn silage + 10% SMS), T2 (80% corn silage + 20% SMS), and T3 (60% corn silage + 40% SMS)

Means with different superscripts within the same column differ, *p* < 0.05

### Biogas and ammonia emissions

The gas volumes were not significantly affected by the inclusion of SMS (Table 4). While CO_2_ production was not influenced, all SMS treatments significantly reduced CH_4_ production (*P* < 0.001), NH_3_ (*P* = 0.005), and H_2_S (*P* = 0.002) compared to the control.

**Table 4:** Effects of partial replacement of corn silage with varying proportions of spent mushroom substrate on gas volume (mL/g substrate) and productions of CH_4_ (mg/g DM), CO_2_ (mg/g DM), NH_3_ (mmol/g DM), and H_2_S (mmol/g DM)

Treatments^1^	Gas volume	CH_4_	CO_2_	NH_3_	H_2_S
Control	3196	65.6^a^	258.9	1025^a^	7828^a^
T1	3056	14.4^b^	137.7	420^b^	2332^b^
T2	3325	21.5^b^	198.1	563^b^	1817^b^
T3	3298	19.2^b^	172.7	578^b^	3458^b^
SEM	189.7	4.49	32.79	117.1	1104.1
*P*-value	0.746	< 0.001	0.080	0.005	0.002

*CH*_*4*_ methane, *CO*_*2*_ carbon dioxide, *NH*_*3*_ ammonia, *H*_*2*_*S* hydrogen sulfide

^1^Treatments included: Control (100% corn silage), T1 (90% corn silage + 10% SMS), T2 (80% corn silage + 20% SMS), and T3 (60% corn silage + 40% SMS)

Means with different superscripts within the same column differ, *p* < 0.05

## Discussion

### Nutrient concentration

Replacement of corn silage with SMS led to marked shifts in the chemical composition of the diets. The lack of a clear difference in DM and OM between T2 and T3, despite their greater contrast in formulation, is likely related to an increase within-treatment variability and the indirect calculation of OM. It is important to note that the values reported represent the analyzed composition after 42 days of ensiling, rather than calculated mixture proportions and therefore reflect fermentation-induced changes in the chemical composition. At higher SMS inclusion levels, the greater ash contribution from SMS and potential non-linear interactions during ensiling may have offset proportional changes in ingredient composition, resulting in convergent OM values in T2 and T3 (Anotaenwere et al. 2024). In contrast, non-structural carbohydrates declined in T1 and T3, reflecting the partial substitution of starch-rich corn silage with a more fibrous co-product.

The inclusion of SMS led to clear increases in fiber fractions. Neutral detergent fiber, ADF, and ADL did not increase in a strictly linear fashion from T1 to T3, reflecting the fact that these were ensiled mixtures rather than simple proportional blends. During mushroom cultivation, white-rot fungi such as *P. ostreatus* selectively and heterogeneously degrade lignin, modifying the lignocellulosic matrix (Wuaku et al. 2025). Subsequent ensiling of the corn silage-SMS mixtures can further alter fiber solubility and partitioning, leading to non-linear responses in fiber fractions across inclusion levels. Similar non-proportional behavior of fiber components has been reported in other studies using fungal-treated or ensiled agro-industrial by-products (Martín et al. 2023). Therefore, apparent discrepancies between pre-fermentation calculated values and post-ensiling analyzed values are expected and do not indicate experimental error.

These compositional changes provide a nutritional basis for the differences in rumen fermentation characteristics and gas production observed in the present study. In particular, moderate SMS inclusion levels (10–20%) appear to improve fiber availability through partial fungal delignification, whereas the highest inclusion level (40%) increases lignin concentration to an extent that may restrict fermentability and microbial access to structural carbohydrates.

### pH, effluent volume and digestibility

The activity of microbes for fermentation depends largely on the pH of the rumen because cellulolytic bacteria present in the rumen are highly sensitive to changes in pH. The pH of the rumen fluid and artificial saliva was recorded as 7.16 when the study commenced. The final values of pH observed in this study (ranging from 7.32 to 7.45) were not significantly affect the inclusion of SMS. Values for pH obtained in this study remained within the ideal range (5.0 to 7.5) for rumen microbial activity (Kumar et al. 2013). This could be a result of the artificial buffer that was constantly supplied in the RUSITEC system. Stable values similar to those obtained in this study have been documented in other studies which involved the use of fungal-treated agricultural residues. Supapong et al. (2019) observed rumen pH (6.9 to 7.1) that was stable when fungal-treated rice straw was introduced to the diets of ruminants. According to Zuo et al. (2018), fungal treatment of corn stover by *Pleurotus* spp. had no significant impact on pH during fermentation and when ensiled. Interestingly, values recorded in the present study were higher than those typically found in studies that used live animals, where the pH of the rumen ranged from 6.0 to 6.8, and vary based on nutrition, frequency of feeding, and production process (Nazli et al. 2018; García-Chávez et al. 2022). This difference is likely due to the buffer, which is in constant supply in the in vitro systems thereby lacking absorption pathways. This leads to the prevention of acid buildup and maintains a more alkaline environment than in live animals. In systems like batch culture or RUSITEC, buffering actions reduce changes in pH made by different treatments. Despite differences in levels of carbohydrate-rich silage or treated forage, Mikołajczyk et al. (2020) stated that there was no significant impact on rumen pH based on the controlled condition of the system. These results support that SMS is a suitable alternative in corn silage-based diets, without increasing the risk of rumen acidosis.

The volume of effluent is usually dependent on the artificial saliva added, composition of the substrate, and varying activities of microbes. In this study, these volumes varied from about 525 to 558 mL across all treatments. T2 had a higher volume of effluent compared to the T1 diet suggesting a rise in fermentation and solubilization of dietary fiber, possibly due to the distinctive characteristics of SMS at this level of inclusion. Martín et al. (2023) reported that SMS consists of partially degraded lignocellulosic material along with residual fungal enzymes. In RUSITEC, these components clearly enhance microbial access and aid in the disintegration of the cell wall components. When fermentation efficiency is improved, a more liquid phase of metabolic byproducts and fluid turnover is attained (Czerkawski and Breckenridge 1977). The treatment T3 (40% SMS) had a volume of effluent that was lower than T2, which may be linked to the increase in the content of lignin that limits water-holding capacity and the breakdown of fiber (Mertens 1997). According to Van Soest (1994), residues that have high lignin are known to hinder microbial activity in the rumen by forming physical barriers that resist the hydrolysis of enzymes.

Even with minimal SMS inclusion, the flow of liquid remained unaffected, as shown by the comparable volume of effluents from the control and T2 groups. This observation is supported by the absence of significant differences in key fermentation indicators, including total volatile fatty acid production and gas output, suggesting that overall microbial activity was maintained. This could support fermentation dynamics similar to those of high-quality corn silage. Additionally, the slight difference between the Control and T3 groups suggests that inclusion of SMS at levels up to 40% SMS has little impact on rumen simulation fluid dynamics. Although minor numerical declines in some parameters may reflect marginal reduction in degradation efficiency, the stable effluent output, together with consistent fermentation end-products, indicates steady microbial activity and system hydraulics, which are key signs of proper fermentation in continuous culture systems. These findings are consistent with a RUSITEC study using dietary additives such as biochar, which reported stable effluent volume despite alterations in gas and CH_4_ emissions (Teoh et al. 2019). The increased volume of effluent observed in T2 further coincided with enhanced fermentation responses, suggesting more active microbial turnover. This effect may be attributed to SMS containing residual fungal enzymes and partially degraded fiber fractions, which can enhance substrate accessibility and microbial degradation rates, as previously reported (Royse et al. 2017).

Organic matter digestibility also remained stable, with values from 86.9% (T2) to 90.8% (T1). This shows consistent microbial breakdown of the OM fraction across treatments. The stability in OMD indicates that including SMS up to 40% did not weaken the fermentative ability of the rumen microbes. This finding agrees with earlier in vitro studies where fungal-treated agricultural residues maintained high digestibility because fungal enzymes partially broke down lignocellulose (Kholif et al. 2022). SMS obtained from *P. ostreatus*, contains residual lignocellulolytic enzymes and pre-digested fibers that likely aid in the colonization of microbes and substrate breakdown in the rumen environment (Royse et al. 2017). The stable OMD results imply that the digestible part of the OM, including carbohydrates and protein, stayed accessible across all treatments. The slight drop in OMD indicated with higher SMS inclusion (T2 and T3) might be due to a higher proportion of indigestible residues, like lignin, which makes up part of the OM but is not broken down by microbes (Van Soest 1994; Mertens 1997). However, these reductions were not statistically significant, suggesting that moderate to high levels of SMS inclusion do not affect OM utilization in simulated rumen conditions. Notably, previous in vivo studies have also backed up these findings. For example, a study by Lu et al. (2024) showed that Hu sheep fed diets with 10% SMS had better nutrient digestion and feed efficiency.

Conversely, DMD demonstrated a highly significant treatment effect. The treatments with 10% and 20% SMS (T1 and T2, respectively) produced the highest DMD values of 46.8% and 48.1%, whereas the control and 40% SMS (T3) showed lower values at 41.1%. This increase at moderate SMS levels probably results from residual fungal enzymes and partially broken-down fibers, which improve overall breakdown of substrate (Olagunju et al. 2023). Similarly, when a live Hu sheep was used to conduct a study by Lu et al. (2024), result showed that a diet with 10% SMS from *P. ostreatus* greatly had an improved nutrient digestibility and feed efficiency. However, increasing the quantity of SMS by more than 10% actually decreased digestibility and changed serum biochemical indicators. Above this level, the nutritional benefits may slow down or even decrease as digestible starch and sugars are replaced with larger or less digestible fungal residues (Olagunju et al. 2023; Lu et al. 2024). It is also important to note that the comparatively lower DMD values relative to OMD reflect the inclusion of indigestible ash and non-degradable components in DMD calculations, whereas OMD excludes mineral fractions, which inherently results in higher OMD values. It should be noted that in RUSITEC systems, DMD reflects the disappearance of total substrate mass, including ash and other non-degradable components, whereas NDFD and ADFD represent the relative degradation of specific fiber fractions; therefore, higher fiber digestibility values compared with DMD can occur without violating fermentation principles (Shaw et al. 2023).

### Fiber fraction digestibility

SMS incorporated into corn silage significantly improved NDFD compared to the control group. This finding is in line with previous studies indicating that SMS derived from *P. ostreatus* contains residual ligninolytic enzymes that may contribute to fiber modification and enhanced microbial access to cell wall components. Although Lu et al. (2024) did not observe statistically significant improvements in fiber digestibility, their study reported numerical trends and shifts in rumen microbial communities that suggest potential indirect effects of SMS on fiber utilization. Direct evidence for improved fiber breakdown through ligninolytic activity has been more consistently demonstrated in in vitro and solid-state fermentation studies involving *P. ostreatus* (Wuaku et al. 2025). However, the 40% SMS inclusion (T3) showed a slightly lower NDFD (63.83%) than T2, suggesting there may be a limit to improving fiber digestibility. The higher NDFD values observed in T1 and T2 could be due to moderate fiber breakdown during mushroom cultivation, which made structural carbohydrates more accessible to the rumen microbes. Similarly, Olagunju et al. (2023) found that solid-state fermentation of corn stover with *P. ostreatus* improved fiber digestibility significantly compared to untreated corn stover. Additionally, in Hu sheep, Lu et al. (2024) discovered that supplementation with SMS mainly when inclusion levels remained at or below 20% also helped to improve rumen fermentation and degradation of fiber. This selective improvement in fiber digestibility reflects partial delignification during mushroom cultivation, which enhances accessibility of structural carbohydrates without proportionally increasing total DMD.

Acid detergent fiber digestibility showed a similar pattern, with T2 having the highest value at 49.3%. These results are similar to the ones obtained in earlier studies that suggest *P. ostreatus* enzymes aid in breaking down the cellulose–lignin complex, making it easier for microbes to access the fiber (Datsomor et al. 2022). Acid detergent lignin digestibility values were highest in the control (25.0%) and T2 (24.9%), with lower values observed in T1 (20.2%) and T3 (19.4%). Although SMS is expected to enhance lignin degradation due to fungal delignification, the lower ADLD in T1 and T3 suggests that some lignin fractions may have become more resistant or that pre-digestion by fungi used up the more easily degradable portions, making them less available to rumen microbes (Olagunju et al. 2023). Consequently, improvements in NDFD and ADFD relative to DMD do not indicate inconsistency, but rather reflect differences in how individual fiber fractions respond to fungal pre-treatment compared with whole DMD degradation. This agrees with findings from Zadrazil (1997), who suggested that fungal degradation of hemicellulose and NDF during SSF could reduce the availability of easily digestible components for subsequent microbial fermentation.

### Volatile fatty acid profiles

Microbes in the rumen of ruminants break down carbohydrates, converting them to VFAs, CO_2_, CH_4_, NH_3_, and microbial biomass (Boadi et al. 2004). Volatile fatty acids are the main end-products of ruminal fermentation and are crucial to ruminant energy metabolism, supporting up to 70% of metabolizable energy requirements (Bergman 1990). The proportions of individual VFAs are influenced by composition of substrates, operational conditions, and population of microbes in the anaerobic digestion system (Lukitawesa et al. 2020).

In this study, inclusion of 10–40% SMS in corn silage did not significantly alter total VFA concentration, indicating that the overall fermentative capacity and energy yield of the system were maintained despite partial replacement of corn silage. This stability likely reflects effective microbial adaptation to SMS-derived lignocellulosic substrates under the buffered and controlled conditions of the RUSITEC system. Among the individual VFAs, acetate proportions numerically increased in the T2 treatment (20% SMS; 74.86%), while CH_4_ production was significantly reduced, which can be directly linked to the significantly higher NDFD and ADFD observed in this treatment. Enhanced degradation of neutral and acid detergent fiber increases the availability of fermentable structural carbohydrates, favoring acetate production by fibrolytic bacteria. This apparent discrepancy indicates that CH_4_ formation was not directly proportional to acetate concentration. Methane production is more closely related to hydrogen availability and methanogenic activity than to absolute acetate levels. The reduction in CH_4_ observed in T2 may therefore reflect a shift in hydrogen utilization toward alternative sinks, such as microbial biomass synthesis or other reduced end-products, rather than changes in acetate production per se (Ungerfeld 2020). Thus, the higher acetate level in T2 reflects more efficient fibrolytic fermentation rather than a general increase in fermentative intensity. From an energetic perspective, acetate is primarily derived from the fermentation of structural carbohydrates such as cellulose and hemicellulose, and serves as a precursor for de novo fatty acid synthesis, particularly in the mammary gland (Yu et al. 2024). Therefore, the acetate response observed in T2 suggests that moderate SMS inclusion may improve the utilization of structural carbohydrates without compromising overall energy availability.

Propionate is the major glucogenic VFA in ruminants and is crucial for hepatic gluconeogenesis and glucose homeostasis. It serves as a hydrogen sink during ruminal fermentation, thereby reducing hydrogen availability for methanogenesis and contributing to lower CH_4_ emissions (Pereira et al. 2022). Proportions of propionate ranged from 13.05 to 15.72%, remained statistically unchanged. In this study, the maintenance of stable propionate levels alongside increased fiber digestibility in T2 indicates that the energetic efficiency was preserved which implies the potential for the mitigation of CH_4_ without adversely affecting the balance between lipogenic and glucogenic energy sources in the rumen.

The A:P ratio is a widely recognized indicator of ruminal fermentation and nutrient utilization efficiency. Low A:P ratio favorable production of propionate, which acts as a hydrogen sink, reducing CH_4_ formation and improving energy efficiency. In the present study, T2 group had the highest A:P ratio (5.81), reflecting its elevated acetate proportion and enhanced fiber fermentation. Higher A:P ratio generally indicate increased cellulolytic activity and fiber digestion, which can also divert hydrogen away from propionate, potentially increasing CH_4_. Despite these variations, A:P values remained within the typical physiological range. Approaches that boost propionate production while maintaining fiber digestion such as adding moderate amounts of bioactive substrates like SMS can improve ruminant productivity and environmental sustainability.

Valerate proportions showed significant differences among treatments. T1 (10% SMS; 2.58%) had the highest value Valerate is mainly produced through the deamination of branched-chain amino acids, which is linked to microbial protein synthesis and nitrogen metabolism (Mitchell et al. 2023). A report from an in vitro experiment by Anotaenwere et al. (2024) stated that ensiling of SMS for 42 d decreased total VFA in diets containing SMS at 10 and 30%, while increasing total VFA concentration in diets containing SMS at 40%.

Other VFAs, such as isobutyrate, butyrate, and isovalerate, did not show any significant differences, suggesting that the overall fermentation environment remained stable across all treatments. This aligns with a study by Mikołajczyk et al. (2020), who demonstrated in an in vitro study that, without negatively affecting VFA profiles, agro-industrial byproducts such as fungal-treated residues can sustain fermentation stability.

### Gas production and greenhouse gas emissions

Values obtained for total gas did not differ significantly across the diets, with values ranging from 3056 to 3325 mL. This shows that the inclusion of SMS did not affect the stability of microbial fermentation kinetics. A previous study conducted by Anotaenwere et al. (2024) reported that adding *P. ostreatus* SMS to corn silage, even at up to 50% inclusion level, did not affect in vitro gas production. This shows a similar fermentative attribute with the control diet. The absence of significant differences in total gas production despite marked changes in dietary composition suggests that the RUSITEC system maintained overall fermentation stability; however, the quality of fermentation differed among treatments as a function of nutrient composition. Diets containing 10–20% SMS increased fiber fractions with residual fungal enzymes and partially degraded lignocellulose, resulting in enhanced DM and fiber digestibility without increasing total gas production (Wuaku et al. 2025). In contrast, 40% SMS diet, characterized by higher lignin content and lower non-structural carbohydrate concentration, had lower digestibility, indicating that higher SMS inclusion may limit fermentative efficiency despite maintaining stable gas volumes (Anotaenwere et al. 2024).

The mitigation of greenhouse gases, particularly CH_4_, NH_3_, and H_2_S was markedly enhanced by the inclusion of SMS in corn silage-based diets. Moderate SMS inclusion levels (10–20%) enhanced fiber digestibility while simultaneously suppressing CH_4_, NH_3_, and H_2_S production, indicating a more efficient ruminal fermentation with reduced energy and nitrogen losses. Among all treatments T1 (10% SMS) exhibited the greatest reduction in CH_4_ output (14.40 mg CH_4_/g DM), corresponding to a 78% decrease compared to the control (65.6 mg/g DM). This pronounced reduction may be attributed to the combined effects of SMS physical structure and biochemical composition of SMS. Increasing SMS inclusion modified the proportions of structural carbohydrates, CP and readily fermentable substrates, thereby altering hydrogen availability and microbial metabolic pathways. The non-linear response of CH_4_ production to increasing SMS levels suggests that low SMS inclusion exerts antimethanogenic effects, potentially mediated by residual fungal metabolites, whereas higher inclusion rates increase fermentable fiber supply, partially counteracting CH_4_ suppression by stimulating hydrogen production. The presence of partially degraded lignocellulosic fibers and residual fungal enzymes likely enhanced microbial fermentation capacity, improving ruminal digestion efficiency and redirecting hydrogen utilization away from methanogenesis (Kholif et al. 2022; Olagunju et al. 2023). Moreover, SMS contains bioactive compounds, including phenolics and terpenoids (Zade et al. 2025), which may directly inhibit methanogenic archaea.

Although CH_4_ production was markedly reduced with SMS inclusion, total gas production was not affected, indicating that overall fermentative activity was maintained and that the decrease in methanogenesis likely reflects a shift in hydrogen utilization rather than impaired microbial function. These findings are consistent with previous studies that reduced CH_4_ emissions in diets rich in rapidly fermentable carbohydrates, such as silage, was due to accelerated microbial fermentation and a shift toward propionate-dominated pathways (Cuervo et al. 2025). Similarly, Mahesh and Madhu (2013) reported lower CH_4_ yields in rations containing cereal stovers previously used for mushroom cultivation, attributing the effect to improved degradability and altered rumen microbial dynamics. Mahesh (2012) also observed a linear decline in CH_4_ production in ruminants fed fungal-treated wheat straw, which contained reduced fiber fractions compared to untreated straw, likely resulting in faster passage rates and shortened ruminal retention time, as less fibrous material is more rapidly fermented.

Furthermore, Sallam et al. (2007) and more recently Kholif et al. (2022) suggested that the reduction in CH_4_ may occur indirectly through enhanced digestion of fiber, which reduces feed particles retention time and limits substrate availability for methanogens. Numerical reductions in CO_2_ production were also observed in SMS-based diets, with T1, T2, and T3 exhibiting lower values than the control, most notably T1 (137.7 mg/g DM), representing an approximate 33% decrease. This finding aligns with Liao and Hsieh (2024), who reported that SMS-containing diets slow fermentation and reduce the rate of gas release.

The control group had the highest ruminal NH_3_ concentration (1025 mmol/g DM), whereas SMS-containing diets showed significantly lower values, ranging from 420 mg/L (T1) to 578 mg/L (T3) indicating improved nitrogen utilization efficiency. These reductions may reflect enhanced microbial protein synthesis or shifts in rumen microbial populations driven by SMS-derived bioactive compounds. In a study, Huang et al. (2023) demonstrated that supplementation with fermented *Pleurotus eryngii* SMS significantly altered rumen bacterial community structure and fermentation dynamics in Hu sheep.

Beyond changes in fiber characteristics, the observed reduction in ruminal NH_3_ concentration may also be attributed to bioactive compounds such as phenolics, terpenoids, and condensed tannin-like substances present in *P. ostreatus* SMS (Kumla et al. 2025). Although SMS is not conventionally classified as a tannin-rich feed, residual phenolic compounds derived from lignin degradation and fungal secondary metabolism may exert protein-binding and antimicrobial effects in the rumen (Kumla et al. 2025). Phenolic compounds can form reversible complexes with dietary proteins at ruminal pH, thereby reducing excessive proteolysis and deamination, limiting NH_3_ release while allowing dissociation under acidic post-ruminal conditions, which may enhance intestinal protein availability and nitrogen efficiency.

In addition, mushroom-derived terpenoids and phenolic metabolites may selectively suppress hyper-NH_3_-producing bacteria and rumen protozoa, key contributors to ruminal protein degradation and NH_3_ formation (Kholif et al. 2022). Such microbial modulation likely favors microbial protein synthesis over NH_3_ accumulation. The pronounced NH_3_ reduction at lower SMS inclusion levels (10–20%) suggests an optimal balance between bioactive compound concentration and fermentable substrate availability, whereas the diminished response at 40% SMS may reflect increased lignin-associated nitrogen or reduced protein accessibility. These mechanisms explain the substantial reduction in NH_3_ emissions without compromising OMD, reinforcing the role of SMS not only as a fiber-modifying feed ingredient but also as a functional source of rumen-active phytochemicals that enhance nitrogen efficiency and environmental sustainability.

A significant decline in H_2_S emissions was observed with the inclusion of SMS, with T2 having the lowest concentration (1817 mg/L) compared to the control (7828 mg/L). Velusami et al. (2013) also had an environmental study that demonstrated that SMS heaps could emit substantial quantities of H_2_S, up to 2083 ppm, particularly when moisture levels exceed 65% and physical disturbance occurs. Although conducted under ambient conditions, this research illustrates the potential for sulfur volatilization from SMS under anaerobic environments, including the rumen. Furthermore, diets with high sulfur content have been observed to alter ruminal fermentation pathways by stimulating the population of sulfate-reducing bacteria like *Desulfovibrio*, also increasing the production of H_2_S while concurrently suppressing CH_4_ emissions (Wu et al. 2021). Microbial imbalance results when sulfur levels go beyond nutritional requirements (> 0.4% DM), impacting rumen epithelial integrity (Wu et al. 2021). This aligns with the result from this study that SMS inclusion results in the lowest H_2_S emission.

## Conclusion

This study shows the relevance of adding SMS to corn silage to enhance fiber digestibility and reduce greenhouse gases while preserving fermentation quality. Incorporating 10–20% of SMS improved dry matter and fiber digestibility. These benefits were accomplished without adversely affecting the pH, gas production, or organic matter digestibility. Additionally, all treatments supplemented with SMS reduced methane, ammonia, and hydrogen sulfide productions, especially at the lower inclusion level (10% SMS), making SMS a promising strategy for lowering enteric greenhouse gas emissions. Overall, the findings suggest that SMS, a cost-effective byproduct of the agro-industrial sector, can serve as a sustainable feed additive for corn silage-based diets. To build on these results, future studies should incorporate in vivo trials to evaluate animal performance, economic feasibility, and the long-term impact on rumen microbiota.

## Data Availability

The datasets used and/or analyzed during the current study are available from the corresponding author on reasonable request.
